# Development and evaluation of a measure of treatment knowledge in guided self-help for eating disorders in a sample of healthcare students and professionals

**DOI:** 10.1007/s40519-019-00737-1

**Published:** 2019-06-26

**Authors:** Paul E. Jenkins

**Affiliations:** 1 School of Psychology and Clinical Language Sciences, University of Reading, Earley Gate, Reading, RG6 6AL UK; 2 Oxford Health NHS Foundation Trust, Warneford Hospital, Oxford, OX3 7JX UK

**Keywords:** Guided self-help, Eating disorders, Psychometric evaluation, Questionnaire, Knowledge, Training

## Abstract

**Purpose:**

The current study describes the development of a measure designed to assess treatment-specific competence in guided self-help (GSH) for eating disorders. The aim is to assess healthcare professionals’ understanding of a popular treatment manual and associated material.

**Methods:**

After initial item development from a review of relevant literature, a range of healthcare staff and students (*N* = 127) completed a knowledge questionnaire. From these data, estimates of psychometric properties were made and a subset of the original sample completed the measure again after 6 weeks.

**Results:**

The final questionnaire consists of 40 items, demonstrating acceptable content validity, internal consistency, and reliability. Significant differences in the number of questions answered correctly were observed between experts in GSH and those with less experience.

**Conclusions:**

This questionnaire offers a means of assessing therapist knowledge of GSH which demonstrates good psychometric properties. Further testing of this instrument is required to establish its full applicability.

**Level of evidence:**

Level IV.

**Electronic supplementary material:**

The online version of this article (10.1007/s40519-019-00737-1) contains supplementary material, which is available to authorized users.

## Introduction

Guided self-help (GSH) based on cognitive behaviour therapy (CBT) principles is a recommended first step in the treatment of the two most common eating disorders: bulimia nervosa and binge-eating disorder (e.g. [1–3]). The premise is that a healthcare professional acts as a facilitator (this term is often used along with ‘therapist’, ‘coach’, and ‘guide’), empowering the patient to use a manualised treatment to solve their difficulties [4]. An explicit manual is used by both patient and facilitator, although adequate knowledge and competence on behalf of the facilitator are likely required to deliver the best possible support (e.g. [5]).

It is self-evident that healthcare professionals should be competent to deliver psychological interventions. Williams and Martinez [6] argue that the qualifications and professional background of the facilitator are less important than their therapeutic competence. Within eating disorders, it has been suggested that “achieving competency requires familiarity with a body of scientific knowledge covering areas that are not generally a part of clinical training” ([7], p 1429) and that the therapeutic skills required “may be more demanding” (p 1455) than in other areas of mental health. As such, although a number of approaches to evaluating competence in individual CBT have been described [8], these are typically difficult to apply to eating disorders given the limited coverage of disorder-specific strategies [9]. This shortcoming has led to the development of a measure of therapist competence in eating disorders (see [9, 10]) although this concerns only a specialised form of CBT, CBT-E (see [11]), which is recommended when GSH is ineffective or contraindicated (e.g. [2]). As the treatments differ, so too does the skill set of a therapist competent in their respective delivery and so therapist competence in CBT-E is not equivalent to competence in GSH. Greater research into competence in GSH is likely to expand attempts to train and evaluate facilitators, as well as improving the quality of the treatment provided. For example, given a growing literature showing that therapists commonly ‘drift’ away from established techniques in CBT [12], the use of knowledge measures may reduce the “idiosyncratic and variable implementation [of GSH] by different providers” that is commonly seen ([4], p 353).

Despite advantages over alternative treatment approaches in terms of flexibility and cost-effectiveness [4], access to GSH remains limited [13, 14]. Although innovative means of delivery have been developed, such as self-monitoring through a smartphone application [13] and providing GSH via the Internet (e.g. [3, 14]), involvement of a clinician with some training in GSH and eating disorders is often required and typically associated with better outcomes than self-help not involving personal communication [15].

The current study aims to describe the development and preliminary psychometric properties of a measure of facilitator competence in GSH, based on early work in this field [16, 17]. The main aim is to present a formal, yet accessible, means of assessing facilitator knowledge of GSH. Description of the initial development of the measure is described, followed by a study aiming to establish preliminary utility and psychometric properties.

## Methods

### Initial questionnaire development

A knowledge questionnaire was designed following the recommendations of Rust and Golombok [18] for situations where no relevant questionnaires exist. The suggestions of Case and Swanson [19] were also useful in designing and evaluating questions. Although there are flaws in Alternate Choice items, this format was chosen given the importance of assessing explicit knowledge of the treatment manual ([7, 10]; see also [20]).

The first stage involved reviewing a treatment manual (in this case Overcoming Binge Eating, 2nd edition [21]) and constructing approximately 50 questions to maximise content validity. Content was loosely organised according to the chapters of the manual and the associated therapist manual [22], although some questions were researched (e.g. in scientific journals) to confirm statements. A similar questionnaire, developed by Carter [16] for evaluating the knowledge of patients treated with GSH (using an earlier edition of the treatment manual), was also reviewed and some items were used and modified, following permission from the instrument’s developers. Through an iterative process of review with other healthcare professionals and a professor of eating disorders with experience in questionnaire design and therapist training, questionnaire length was reduced. Four pilot subjects completed the first draft of the questionnaire (47 items) and provided feedback to assess both clarity and level of difficulty of the items. One item was removed due to lack of clarity and inconsistent empirical findings. Subsequently, five individuals with significant experience in GSH independently assessed the remaining items for representativeness (on a scale of 1–4) and also commented on clarity. This process was conducted at an early stage to eliminate items felt to be unrepresentative and to enhance face validity (see [23]). Items coded by at least 4 of 5 experts as either not relevant or somewhat relevant (scores of 1 and 2 respectively) were deleted, a variation of the method of Zaichkowsky [24]. One item was deleted following this procedure, leaving a 45-item measure.

The questionnaire consists of three parts and takes around 10–15 min to complete. Part 1 aims to evaluate knowledge of the treatment manual, predominantly the educational component of Overcoming Binge Eating. Part 2 is designed to assess knowledge of the treatment itself and was based in part on the facilitator’s manual for GSH [22]. Part 3 makes use of two clinical vignettes and aims to provide a ‘real world’ scenario to evaluate knowledge, with clinical descriptions of bulimia nervosa and binge-eating disorder. Answers for Parts 1 and 2 are Alternate Choice Items, with Multiple Choice Items used for the vignettes. A response of “don’t know” is included for the Alternate Choice items to reduce guessing ([16]; see also [25]).

### Participants and design

Participants were recruited from two pools to maximise potential generalisability of the results, targeting individuals representative of those who might provide GSH in clinical settings (i.e. purposive sampling). A power analysis based on the ability to detect medium-sized correlations (*r* = 0.3, two-tailed) suggested a minimum sample size of 84 [26]. Conduct of the study was approved by the Research and Development department of Oxford Health NHS Foundation Trust and the University of Reading School of Psychology and Clinical Language Sciences Research Ethics Committee. Informed consent was obtained from all individual participants included in the study.

#### Sample 1

All members of staff working for one of the three eating disorders services or one of two general mental health services (within one NHS Trust in England) were posted hard copies of the questionnaire to their work base and asked to return responses within 6 weeks. An information sheet was also provided. Staff lists for the eating disorders service were consulted and questionnaires distributed to 98 staff. The general mental health services were contacted by a member of the Trust’s Research and Development team and the total number of staff approached is not known (but is likely to number under 60 individuals). Following this, several participants asked to provide responses electronically; some requested and completed an electronic version and others took photographic images and attached these to an e-mail. E-mail reminders were sent after 2 and 4 weeks to all staff, after which few additional responses were received. In total, 77 members of staff provided questionnaires, including both qualified (e.g. nurses, clinical psychologists, psychiatrists) and non-qualified healthcare professionals (e.g. healthcare assistants). Participants were asked to provide authentic responses to the questions, although it was not possible to monitor whether they consulted resources (e.g. the Internet) when completing the measure. The sample was predominantly female (*n* = 65; 84.4%; data missing from one individual) with a mean (SD) age of 35.8 (10.7) years. Self-reported experience working with patients with eating disorders ranged from 0 to 20 years (mean [SD] = 3.48 [4.90]).

#### Sample 2

Postgraduates studying areas related to clinical psychology at a UK university and staff working for another mental health service were approached in person and asked to complete hard copies of the measure. Procedures differed slightly from those involving Sample 1 in that students were approached during lectures and asked to complete the questionnaire during breaks and staff were asked to return the questionnaires to a researcher in person; no e-mail reminders were sent. In total, 50 individuals provided questionnaires, also predominantly female (*n* = 44; 88.0%) with a mean (SD) age of 24.1 (2.78) years. Self-reported experience was lower (due to the majority of students), with a range from zero to one-and-a-half years (mean [SD] = 0.12 [0.34].

#### Total sample characteristics

Mean age was 30.8 years (SD = 10.11) and most participants (86.5%) identified as female. Self-reported experience was a mean of 2.11 years (SD = 4.12). For neither sample was it possible to accurately determine response rate, although it is estimated that around 40% of those approached returned questionnaires, consistent with studies of this type. As noted above, data from both samples were pooled given the applicability to those likely to deliver GSH.

### Item analysis

Due to the structure of the responses, every item was coded as “1” for a correct answer and “0” if an incorrect or “don’t know” response was given, providing a total score as the sum of all responses (maximum = 45). The facility level of items (the ratio of the number of respondents who give the wrong response to the number of total respondents; [18]), which ranges from 0.0 to 1.0, is presented. The ideal range of the facility index should be between 0.25 and 0.75, with an average of 0.5 for the entire questionnaire [18] although this varies according to the design aims of the test. The related item variance statistic is also presented, calculated with the following formula [18], where *p* is the facility index:

$${\text{Item variance}}\, = \,p \times \, \left( {1{-}p} \right).$$

Item variance cannot (mathematically) exceed 0.25 (see [18] for details) and clustering as close to 0.25 as possible is ideal, as variance rapidly declines with more extreme facility indices. This was used as the primary method for item deletion, in order to remove items representing common (i.e. not treatment-specific) knowledge. However, the use of such methods “owes as much to common sense and convention as it does to statistics” ([18], pp 58–59) so some items were retained if felt to represent essential knowledge of the treatment.

### Statistical analyses

Descriptive statistics are presented at item and scale level and non-parametric inferential methods were used due to unequal group sizes. Estimates of internal consistency are made using the Kuder–Richardson-20 (KR20) formula, a measure designed for surveys with dichotomous responses; values between 0.70 and 0.90 are generally considered acceptable [27]. This statistic also provides a proxy for split-half reliability (see [27, 28]). Corrected item-total correlations (the correlation between each individual item and the total score minus that item) are obtained, with values outside of the range 0.20–0.80 indicating concern [18]. Construct validity was assessed with Kruskal–Wallis (*H*) and Mann–Whitney tests (*U*) by comparing the scores between groups hypothesised to differ on knowledge of GSH (less vs. more relevant clinical experience) and where no differences were expected to occur (e.g. by gender). To reduce the impact of sampling error and report uncertainty around point estimates, such as those for reliability coefficients [29], 95% confidence intervals (using bias-corrected Monte Carlo simulation with 10,000 seeds) are reported. Test–retest reliability was assessed through the use of Kendall’s tau (*τ*) and the phi (*φ*) coefficient (an estimate of effect size). Although some cutoffs have been suggested, the stated strength of association is typically lower for *τ*, compared to Spearman’s coefficient (*r*_s_), for example (see [30]) and may provide a more accurate estimate of population means. Data were analysed using SPSS (v24.0, New York, NY, USA) and Microsoft Excel (Redmond, WA, USA).

## Results

### Reliability and validity

Regarding content and face validity, items were based directly on information contained in Overcoming Binge Eating [21], a popular self-help programme for eating disorders. Thirty-nine items (87%) were rated by at least 4 of the 5 experts as scoring ≥ 3 in representativeness (i.e. quite relevant or highly relevant). The KR20 was 0.90 (95% CIs 0.87–0.92), which indicates excellent internal consistency and split-half reliability. A subsample of individuals (*n* = 6) completed the questionnaire on two occasions (6 weeks apart). Overall test–retest reliability was good, *τ* = 0.59 (*φ* = 1.50).

### Item analysis

On an item level, means (i.e. % correct) ranged from 0.12 to 0.96, indicating a good range. No individual answered all questions correctly, with the facility index ranging from 0.04 to 0.88 (mean = 0.39) and item variances from 0.04 to 0.25. 112 individuals (88.2%) completed all items, with omitted items all from the vignettes section (Part 3). Of 5715 possible responses (45 items × 127 respondents), 5630 questions were completed (98.5%). As there is no established procedure for pro-rating this questionnaire, completion of missing data was not considered. Item-total correlations ranged from 0.117 (Item 9) to 0.608 (Item 31).

### Scale statistics after item deletion

**Fig. 1:**
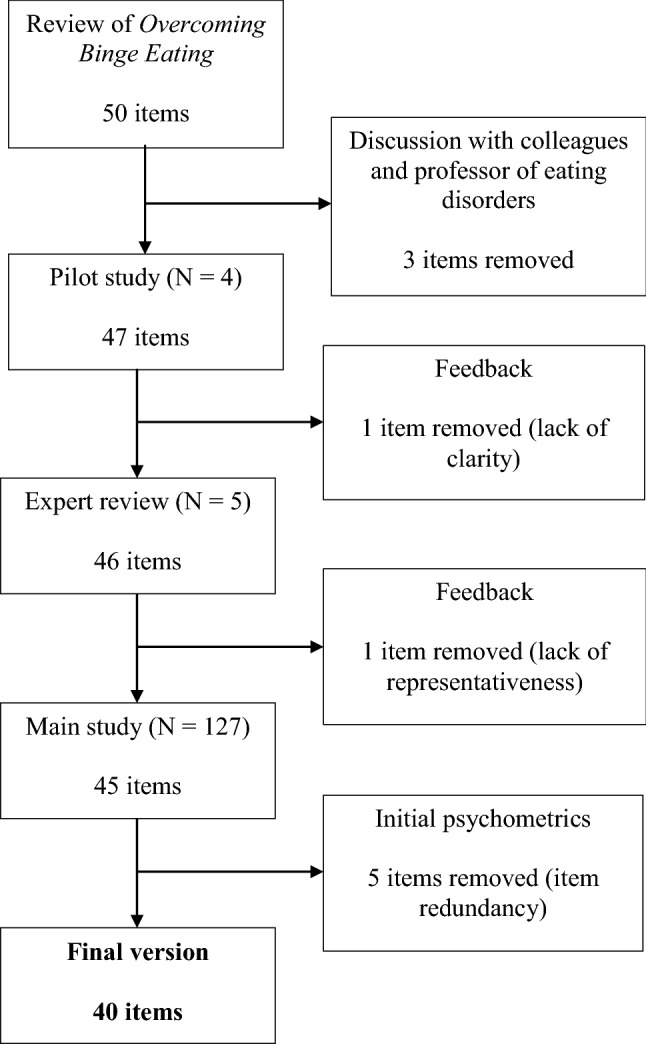
Flowchart of the development and refinement of the measure under study

The process for the refinement of the measure is summarised in Fig. 1. Following review of item-total correlations and facility indices (see Table S1), five items were removed from the scale. The final (40-item) questionnaire (see Online Appendix) retained excellent internal consistency and split-half reliability (KR20 = 0.88; 95% CIs 0.85–0.91). The mean (SD) score for the total sample was 22.24 (7.60) and ranged from 4 to 40. The facility index ranged from 0.11 to 0.888, with an average of 0.44, and the lowest item variance was thus 0.10.

### Known-groups validity

As hypothesised, there were no differences in scores between males and females (*U* = 826.00, *p* = 0.469, 95% CIs 0.460 – 0.479) and the total score was not correlated with age (*r* = 0.072, *p* = 0.446, 95% CIs − 0.122 to 0.255). The overall test for differences between professional groups was not significant (*H* = 5.147, *p* = 0.418, 95% CIs 0.409–0.428). Clinical experience was correlated with total score (*r* = 0.366, *p* < 0.001, 95% CIs 0.246–0.483) and further classified according to four groups (under 1 year; 1–2 years; 2–5 years; more than 5 years). These groups differed significantly (*H* = 24.243, *p* < 0.001), with less experienced individuals (under 2 years) scoring lower than more experienced groups. Similarly, eight individuals with significant experience in GSH were identified as a group known to have greater knowledge of the treatment (‘Specialists’). Total scores from this group were compared to the remainder of the sample and were significantly different (*U* = 27.50, *p* < 0.001). The mean score for Specialists was 35.4 (median = 36). All specialists providing complete data scored above 33, with 3 other individuals (2.52%) scoring at this level.

## Discussion

The current report describes the development of a measure for assessing competence and knowledge of GSH for eating disorders. The questionnaire complements existing measures within eating disorders (e.g. [10]), expanding the portfolio for assessing therapist competence to another level of stepped care (see [2]). The questionnaire showed good reliability, internal consistency, and preliminary validity within this sample, and also has the advantage of being relatively brief. However, more research (e.g. investigating the association between baseline questionnaire scores and patient outcomes) is required to confirm the predictive utility of the measure. It is hoped that the measure could be easily included in protocols using GSH or as an additional element of therapist training (e.g. [13, 14]).

Scores on the questionnaire were significantly higher in groups with greater experience of GSH, although a minority of individuals who reported clinical experience outside of GSH produced high scores. This may reflect the scores of participants who had worked with individuals with eating disorders or attended training courses on CBT for eating disorders (CBT-ED), which has considerable conceptual overlap with GSH. Although the required skill set may differ between GSH and CBT-ED [4], such individuals’ knowledge of CBT-ED may be similar to that required to deliver GSH and thus produce high scores on the measure described here. In line with this, it was found that total scores were correlated (to a moderately strong degree) with experience working in eating disorders. Furthermore, there was some evidence of discriminant validity in that scores on the measure were unrelated to other variables (e.g. age, professional group). The opinions of GSH experts were sought at an early stage, with results indicating good content domain sampling, and good face and content validity (but see [31], for a critique of this methodology). Efforts were made to increase face validity and domain sampling by asking both lay people and experts to comment on earlier versions of the questionnaire. However, as outlined by Beckstead [31], further studies are required to investigate the wider validity of the measure.

Several questions were frequently answered correctly and were removed as they might represent ‘common knowledge’ about eating disorders (e.g. ‘Eating disorders usually have one single cause’; see online supplement). Others were answered correctly by most respondents but were retained as they were thought to assess items of particular importance in the knowledge of prospective GSH therapists (e.g. “It is essential to examine food diaries in every GSH session”). The overall facility index approached the ‘desirable’ average of 0.5 [18] and most questions demonstrated good item discrimination although the absence of “don’t know” responses in Part 3 may have increased the likelihood of guessing and thus affected reliability.

The primary goal was to develop a measure of knowledge of a manualised self-help approach which could be used in the training of self-help providers to identify both gaps in knowledge and degree of ‘readiness’ to begin delivering treatment. As successful provision of self-help is based in part on the therapist’s knowledge of the programme (and associated therapist’s manual) and can occur without formal qualifications, knowledge of the manual is an important element of competence and thus should be evaluated; to the author’s knowledge, no measure currently exists for this purpose. An ongoing goal is for the measure to be used as an aid in supervision of GSH therapists (e.g. see [13]), to identify gaps and areas for development and also as part of research studies. The measure was not designed as a ‘pass/fail’ questionnaire although a score below 32 (80%) might suggest the need for greater preparation before commencing the delivery of support. This cutoff was not determined statistically but represents a convenient threshold, above which all ‘experts’ scored (see above) and only a minority (around 10%) of the remainder of the sample. Future studies could investigate this further, for example, using receiver operating characteristic analysis where groups (e.g. pre- and post-training in provision of GSH) are known. The use of vignettes (using Multiple Choice responses) was designed to include ‘real world’ scenarios and more than just “basic facts” ([8], p 488; see also [5]) although more sophisticated measures, such as role-play, could go further. It is important to note, however, that therapist knowledge alone is not sufficient for successful delivery of GSH [32, 33], but is one component of overall therapist competence. The measure detailed here is proposed as an important tool, upon which therapist competence may be further developed by supervision and direct feedback (e.g. [34]).

### Strengths and limitations

Development of this measure represents an important step in the evolving field of therapist competence in eating disorders (see [5]) and is the first to the author’s knowledge looking at competence in GSH. Recommended steps for novel questionnaire design [18] were followed and psychometric properties appear promising. However, the sample was limited in size (particularly for analysis of sub-groups) and it was not possible to determine how many individuals approached for the study did not participate. The questionnaire was evaluated on both healthcare professionals and healthcare graduates as these represent individuals commonly providing GSH (e.g. see [4]), although methods of recruitment differed between samples which may have introduced bias. Furthermore, there may have existed differences in treatment knowledge between respondents and non-respondents which could have influenced findings and reflected a non-response bias, perhaps in terms of motivation to participate. However, given the range of responses obtained, the current sample was likely sufficient to establish the initial psychometric properties of the measure. To preserve anonymity and blindness to the investigator, self-reported levels of experience were obtained, precluding any formal evaluation of experience or competence, which was a shortcoming of the study. Larger samples are required to investigate psychometrics more fully, which were limited in the current study by the relative novelty of the measure and lack of access to large numbers of trained (and yet-to-be-trained) GSH therapists. Such work would also be helpful in determining whether fewer items could be included and whether any scales emerge from the full questionnaire, for example by conducting further analyses (factor analysis can be problematic with dichotomous responses [27]). The knowledge questionnaire also pertains to one GSH treatment manual, although a number of alternatives exist. Whilst some knowledge (e.g. Part 1) may be common to all manuals, some elements of the questionnaire may apply only to the content of Overcoming Binge Eating.

Turning to the potential use of the questionnaire, it is inevitable that practice effects may occur with frequent use, particularly if given appropriate supervision and if the questionnaire is used as an ongoing evaluation of competence (e.g. [35]). Furthermore, as a version of the questionnaire with the ‘correct’ answers is available (upon request from the author), it is feasible that this could be used to inflate knowledge of the questionnaire rather than GSH treatment itself. However, the goals of the questionnaire are to provide a baseline for establishing training needs and identifying gaps, and the specialist group in the current study did not all score at ceiling.

## Conclusions

The current study describes the development of a questionnaire assessing knowledge of a popular treatment manual for recurrent binge eating. Initial psychometric properties are described, although larger datasets are required to endorse these findings, and the author would welcome data from other parties; information about the effects of training would be particularly desirable. It is hoped that the measure might be used on an individual level to assess knowledge, relate this to therapist competence, and to help train therapists in the provision of GSH for eating disorders. It might also be used to study the relationship between therapist knowledge and outcome in GSH treatment studies and, thus, support efforts to increase adherence and scalability of such treatments [36].

## Electronic supplementary material

Below is the link to the electronic supplementary material.

Supplementary material 1 (DOCX 13 kb)

Supplementary material 2 (DOC 70 kb)
