# Supplementary material for: Development and evaluation of a measure of treatment knowledge in guided self-help for eating disorders in a sample of healthcare students and professionals
Source: Eat Weight Disord. 2019 Jun 26;25(4):833–9. doi: 10.1007/s40519-019-00737-1 (PMC7399678; doi:10.1007/s40519-019-00737-1)
Supplement: Supplementary file 1 — Supplementary material 1 (DOCX 13 kb) [file 40519_2019_737_MOESM1_ESM.docx]

Supplementary Table 1. Item difficulty and item-total correlations (see text for details)

| Item number | Facility Index | Item Variance | Item-total correlation |
| --- | --- | --- | --- |
| 1 | 0.134 | 0.116 | 0.227 |
| 2* | 0.079 | 0.073 | 0.167 |
| 3 | 0.134 | 0.116 | 0.443 |
| 4* | 0.039 | 0.038 | 0.231 |
| 5 | 0.165 | 0.138 | 0.151 |
| 6 | 0.283 | 0.203 | 0.269 |
| 7 | 0.205 | 0.163 | 0.316 |
| 8* | 0.094 | 0.086 | 0.294 |
| 9 | 0.134 | 0.116 | 0.117 |
| 10 | 0.425 | 0.244 | 0.511 |
| 11 | 0.315 | 0.216 | 0.468 |
| 12 | 0.669 | 0.221 | 0.302 |
| 13 | 0.520 | 0.250 | 0.400 |
| 14* | 0.063 | 0.059 | 0.339 |
| 15 | 0.370 | 0.233 | 0.39 |
| 16 | 0.213 | 0.167 | 0.269 |
| 17 | 0.433 | 0.246 | 0.289 |
| 18 | 0.110 | 0.098 | 0.404 |
| 19 | 0.110 | 0.098 | 0.198 |
| 20 | 0.543 | 0.248 | 0.373 |
| 21 | 0.772 | 0.176 | 0.521 |
| 22* | 0.071 | 0.066 | 0.179 |
| 23 | 0.843 | 0.133 | 0.337 |
| 24 | 0.331 | 0.221 | 0.430 |
| 25 | 0.638 | 0.231 | 0.566 |
| 26 | 0.819 | 0.148 | 0.459 |
| 27 | 0.331 | 0.221 | 0.396 |
| 28 | 0.472 | 0.249 | 0.550 |
| 29 | 0.748 | 0.188 | 0.582 |
| 30 | 0.567 | 0.246 | 0.314 |
| 31 | 0.559 | 0.247 | 0.608 |
| 32 | 0.512 | 0.250 | 0.403 |
| 33 | 0.661 | 0.224 | 0.428 |
| 34 | 0.598 | 0.240 | 0.461 |
| 35 | 0.315 | 0.216 | 0.391 |
| 36 | 0.244 | 0.185 | 0.454 |
| 37 | 0.386 | 0.237 | 0.436 |
| 38 | 0.559 | 0.247 | 0.567 |
| 39 | 0.283 | 0.203 | 0.470 |
| 40 | 0.885 | 0.102 | 0.426 |
| 41 | 0.265 | 0.195 | 0.170 |
| 42 | 0.464 | 0.249 | 0.212 |
| 43 | 0.589 | 0.242 | 0.429 |
| 44 | 0.402 | 0.240 | 0.144 |
| 45 | 0.411 | 0.242 | 0.145 |

*Note: Item deleted at final stage
