# Supplementary material for: Development and evaluation of a measure of treatment knowledge in guided self-help for eating disorders in a sample of healthcare students and professionals
Source: Eat Weight Disord. 2019 Jun 26;25(4):833–9. doi: 10.1007/s40519-019-00737-1 (PMC7399678; doi:10.1007/s40519-019-00737-1)
Supplement: Supplementary file 2 — Supplementary material 2 (DOC 70 kb) [file 40519_2019_737_MOESM2_ESM.doc]

Part I. Knowledge of the treatment manual

|  | **True** | **False** | **Don’t Know** |
| --- | --- | --- | --- |
| 1. Loss of control distinguishes binge eating from everyday overeating |  |  |  |
| 1. Having been diagnosed with anorexia nervosa as a teenager means that you cannot meet criteria for a diagnosis of bulimia nervosa as an adult |  |  |  |
| 1. Dieting characterised by strict rules and ‘all-or-nothing’ thinking can create a cycle of binge eating and dieting |  |  |  |
| 1. Self-induced vomiting maintains binge eating |  |  |  |
| 1. Drinking large amounts of water to aid vomiting is relatively risk-free |  |  |  |
| 1. Excessive binge eating can have dangerous effects on the stomach |  |  |  |
| 1. Swelling of the salivary glands is one of the physical effects of regular self-induced vomiting |  |  |  |
| 1. Taking laxatives at, or above, the recommended dose eliminates most of the calories consumed during a binge |  |  |  |
| 1. A family history of obesity increases the risk of bulimia nervosa and binge eating disorder |  |  |  |
| 1. Eating disorders tend to run in families |  |  |  |
| 1. Only a minority of individuals with binge eating problems can be treated as outpatients |  |  |  |
| 1. An accompanying clinical depression can interfere with progress during guided self-help treatment |  |  |  |
| 1. One advantage of guided self-help over therapist-led cognitive behaviour therapy is that it is inherently more empowering |  |  |  |
| 1. People for whom guided self-help has been ineffective should **not** be offered a more intensive treatment, such as cognitive behaviour therapy or interpersonal psychotherapy |  |  |  |

Part II. Knowing the treatment: how to be an effective GSH therapist

|  | **True** | **False** | **Don’t Know** |
| --- | --- | --- | --- |
| 1. One role of the therapist is to keep the patient motivated |  |  |  |
| 1. The program is “therapist-led”; that is, while patients are encouraged to contribute, the therapist is responsible for determining the content of sessions and setting an agenda |  |  |  |
| 1. GSH sessions are weekly over the full course of treatment |  |  |  |
| 1. It is essential to examine food diaries in every GSH session |  |  |  |
| 1. GSH typically lasts 8 – 16 weeks but a patient’s recovery will usually continue beyond this |  |  |  |
| 1. It is OK for sessions to regularly exceed 20 – 25 minutes |  |  |  |
| 1. As overvaluation of weight and shape is central to eating disorders, this is addressed early on in GSH |  |  |  |
| 1. Patients reporting current intent to end their life should not be offered GSH as a first-line treatment |  |  |  |
| 1. In cases where individuals with eating disorders are severely overweight or obese, they should address weight loss prior to starting GSH |  |  |  |
| 1. There exists good evidence that GSH is appropriate for individuals who purge but do not binge eat |  |  |  |
| 1. It is acceptable for a patient to be receiving psychotropic medication while receiving GSH |  |  |  |
| 1. GSH has a stronger evidence base for binge eating disorder (BED) and bulimia nervosa (BN) than anorexia nervosa (AN) |  |  |  |
|  |  |  |  |
|  | **True** | **False** | **Don’t Know** |
| 1. Individuals who make early change in GSH are more likely to have a better outcome than those who do not |  |  |  |
| 1. GSH has beneficial effects on ED symptoms but not general psychiatric symptoms |  |  |  |
| 1. Signs of deterioration within the first few weeks of treatment indicate that GSH should be stopped immediately |  |  |  |
| 1. If a patient fails to keep an appointment, the therapist should wait at least a week for the patient to make contact before contacting them |  |  |  |
| 1. If a patient is regularly late for sessions, the therapist should enquire about this, and address this with the aid of the book |  |  |  |
| 1. In the event of unexpected weight gain, therapists should suggest that patients weigh themselves more frequently (e.g., twice a week) |  |  |  |
| 1. If a patient is struggling to make change on the first couple of steps of GSH, treatment should move ahead to other parts of the book to see if this helps |  |  |  |
| 1. If a patient has been able to stop binge eating early on in GSH treatment, it is best to continue with the program rather than ending early |  |  |  |
| **For investigator use only** |  | | |
| Score (Part I) |  | | |
| Score (Part II) |  | | |
| Score (Vignettes) |  | | |
| Score (Total) |  | | |

Part III. Clinical vignettes

You are at Session 4/12 (Week 4) with a female referred with bulimia nervosa. You are on Step 2 of Overcoming Binge Eating (2^nd^ edition), and considering moving on to Step 3. She has had some early success in reducing the frequency of objective bulimic episodes and associated self-induced vomiting, and was initially pleased with this. However, at the weekend before the session she went to a party and, having skipped lunch, later ate more than she had planned. She subsequently went home early and began a larger binge, which culminated in vomiting. After briefly discussing this event, what would be the most appropriate next step to take?

- Reiterate the importance of using diaries and how this fits with regular eating;
- Prioritise this issue in the session as it may represent a significant relapse, or decrease in motivation;
- Discuss triggers to binge eating and use chain analysis to understand what caused the binge;
- Use problem-solving to consider alternative strategies that she might have used.

Later in the same session, you establish that she has not monitored her dietary intake for the weekend, but restarted again on Monday. Having discussed the relevant parts of the book, the best approach would be to:

- Remind her of the importance of regular monitoring, and construct the diary in the session, asking her to recall her intake;
- Reconsider moving on to Step 3 as she has not mastered Step 2 yet;
- Encourage her to continue with self-monitoring, even on days where she did not eat what she had planned;
- Play down this part of the session, and concentrate on what she would like to work on.

Towards the end of the session, she reiterates her concern about the weekend’s events. You have had 25 minutes together and she has asked to speak to you more about the weekend’s events. The best approach would be to:

- Allow her an extra 5 minutes to tell you more, and then end the session;
- State firmly, but in a supportive manner, that you have already discussed this and that the sessions need to end on time;
- Ask her to write down what you have already discussed about the weekend, and to discuss with a trusted friend or partner;
- Remind her of the duration of the session and, if appropriate, encourage her to read parts of the book relevant to problem-solving to address the difficulty.

You are working with a male patient with binge eating disorder. He attends Session 2 and reports having struggled to start food diaries as he “doesn’t see the point.” Having briefly discussed the issue, the best approach from the options available would be to:

- Skim the relevant parts of the book together, and ask what he would say to another patient if he were a therapist;
- Reiterate the coverage of Chapters 1, 4, & 5, and review again next week;
- Complete a ‘pros and cons’ of change worksheet to improve motivation;
- Discuss together what the book advises in ‘Getting Ready’ and ‘Starting Well’.

You have worked with the patient to explore the importance of monitoring, but he still appears unconvinced. A helpful approach would be to:

- Use a different type of diary (e.g., Dysfunctional Thought Record);
- Identify in more detail the reasons he found it difficult to monitor, and then address these in the session;
- Complete a day’s monitoring together (e.g., yesterday) and use this to point out the benefits of writing things down;
- Discuss past ‘successes’ with other patients – he may not trust your opinion yet, and might benefit from some concrete examples.

Having spent most of the session discussing the issue, your patient remains unconvinced and resigned to not monitor this week. You should:

- Encourage him to do his best, and discuss more next week;
- Meet again, completing the next session regardless of what he has done;
- Advise that this is not a good idea, and then consider (e.g., in supervision) whether he is ready to engage in treatment at this time;
- Discharge him.

- End -
